# An ongoing WE: A focused ethnographic study of the relationship between child and hospital clown during recurrent pain‐related procedures and conditions

**DOI:** 10.1002/pne2.12005

**Published:** 2019-08-22

**Authors:** Helle Nygaard Kristensen, Erik Elgaard Sørensen, Jennifer Stinson, Helle Haslund‐Thomsen

**Affiliations:** ^1^ Department of Pediatrics Aalborg University Hospital Aalborg Denmark; ^2^ Clinical Nursing Research Unit Aalborg University Hospital Aalborg Denmark; ^3^ Department of Clinical Medicine Aalborg University Aalborg Denmark; ^4^ Lawrence S. Bloomberg Faculty of Nursing University of Toronto Toronto ON Canada; ^5^ Child Health Evaluative Sciences, Research Institute The Hospital for Sick Children Toronto ON Canada; ^6^ Clinic for Anesthesiology Child Diseases, Circulation and Women Aalborg University Hospital Aalborg Denmark

**Keywords:** children, ethnography, hospital clown, pain management, pediatric nursing, procedural pain

## Abstract

**Aim:**

This study explored the interaction between child and hospital clown during recurrent hospitalizations for repeated pain‐related procedures and conditions.

**Background:**

Despite improvements in the management of pain in hospitalized children, procedural pain in particular is a common experience for hospitalized children, and they continue to report undertreated pain. Hospital clowns are widely used as a nonpharmacological intervention in hospitalized children. Little research has examined the influence of hospital clowns during recurrent hospitalizations on repeated painful procedures.

**Design and methods:**

Ethnographic fieldwork using focused ethnography was conducted. Data were collected during October–December 2017 using participant observation and informal interviews with children at one pediatric unit at a Danish university hospital. Data include 61 interactions between children aged 4–14 years and hospital clowns. The participants comprised 13 children undergoing recurrent hospitalizations. The data were coded using thematic analysis, and the research team verified the resulting themes.

**Results:**

The overarching theme was defined as *An ongoing WE*, based on two identified themes, that is, *Stronger in a WE* and *Hope in the WE*. The WE was characterized by a responsive interaction between the child and clown, which evolved over the course of an ongoing relationship.

**Conclusion:**

This study demonstrates how an ongoing WE was constructed with children during repeated painful procedures and conditions. Specifically, the study emphasizes the importance of developing a trusting relationship on the child's terms. Children seemed to experience enhanced coping with painful procedures during the recurring hospital clown encounters, thus reinforcing their competence and hope for coping with future painful procedures. These findings may improve psychosocial care for hospitalized children undergoing repeated painful procedures and conditions and may facilitate multidisciplinary initiatives, such as nurses’ advocacy for the inclusion of hospital clowns during recurrent hospitalizations for repeated painful procedures to ensure optimal pain management.

## 1 INTRODUCTION

Despite improvements in the management of pain in hospitalized children, children continue to report unmanaged pain.^1^, ^2^, ^3^ Procedural pain in particular is a common experience for hospitalized children.^2^, ^3^, ^4^ Research has found high rates of pain among hospitalized children.^2^, ^3^ Children in long‐term treatment for diseases such as cancer must undergo numerous potentially painful procedures related to treatment and care (e.g., venipuncture, nasogastric tube insertion, mobilization).^5^ Many children describe such procedures and treatments as the most painful and distressing part of their disease.^6^, ^7^ Findings suggest that children repeatedly exposed to painful procedures experience more pain and have lower pain thresholds.^8^ Thus, children experiencing untreated pain during repeated painful stimuli and negative previous experiences are at risk of increased levels of pain and negative psychological sequelae for subsequent procedures.^9^, ^10^ Moreover, the child's individual temperament, previous experiences, age and developmental level, and the social and contextual terms such as parent's role may influence the child's perception of pain.^11^, ^12^ Clinical guidelines on pediatric pain management^13^, ^14^ recommend inclusion of both pharmacological and nonpharmacological strategies. Reviews also support various nonpharmacological psychological strategies tailored to the individual child.^15^ Psychological interventions, especially using a multidisciplinary approach, can help reduce children's pain and distress.^15^ Hospital clowns (referred to as clowns) can be seen as a complementary approach in pain management using distraction, humor, and imagery.^16^, ^17^, ^18^ More specifically, clowns create forms of play that invite the individual child into an imaginative and safe relationship, which can support the child in potential stressful situations.^19^, ^20^, ^21^ Previous research 22, 23, 24 on child‐clown interaction during invasive procedures compared with standard care has been inconsistent concerning the impact of clowns on the experience of pain. Recent work by Kristensen et al. 4 showed a significant decrease in pain for acute hospitalized children aged 7–15 years undergoing venipuncture with the presence of a clown. The therapeutic clown role is described as one in which the clown establishes an empowering and supportive relationship with the child.^18^, ^20^ However, little research has explored interactions between clowns and hospitalized children.^18^, ^20^ Most current research focuses on one‐time specific procedures.^19^, ^25^ There is a paucity of empirical evidence on the influence of interactions between children and clowns during recurrent hospitalizations, comprising repeated pain‐related procedures and conditions. Therefore, the present study explored child‐clown interactions during recurrent hospitalizations, comprising repeated painful procedures and conditions.

## 2 METHODS

### 2.1 Study design

An exploratory qualitative approach was used in order to provide knowledge of the interaction between the child and clown. The study design was guided by practical ethnographic principles^26^, ^27^ and focused ethnography.^28^, ^29^ Focused ethnography, which is defined as short‐term and not continual,^29^ is a suitable method when the research question focuses on well‐defined concerns—in this study, the characteristics of the interaction between the child and clown in specific painful situations.

### 2.2 Study participants

The study was performed in a pediatric ward located in a public university hospital in Denmark. The ward admitted hospitalized children with cancer and rheumatological conditions. The clowns were a part of the healthcare team and had collaborated with nurses and doctors for several years before this study was undertaken. The clowns had received a formal education, with training in acting as a hospital clown combined with an understanding of the medical conditions and behaviors characteristic of child patients. The clowns wore a red nose and colorful clothes. Overall, the strategies of the clowns included creating a relationship using a variety of strategies such as cognitive distraction, humor, and imagery, and articulation of the child's expression was a specific part of the approach. The clowns mostly worked together in pairs. They were present from three to four hours a day on weekdays. On a given day, the nurses recorded on a visible board which clowns were present and during what period of time. On the day before an encounter, the nurses informed the clowns about the child's condition, and they planned the visits together based on details such as the scheduled time of the child's procedure and the nature of the procedure. In this study, one clown (ID A) was present during all encounters. Table 4 outlines these encounters, providing demographic information as well as the number and characteristics of the encounters.

Children were included in the study if they had recurrent interactions with the clowns during different pain‐related procedures and conditions (e.g., venipuncture, intramuscular injections, port‐a‐catheter access, nasogastric tubes, mobilization). In addition, encounters without a pain‐related situation were carried out (as a part of establishing a relationship; see Table 4). The children may have had contact with the clowns prior to the study or they may establish contact from the beginning of this study period with the potential to follow the child during recurrent encounters. The presence of at least one parent and the ability to speak Danish or English was required. Children with less than two encounters with clowns and no painful procedures or conditions (e.g., venipuncture, intramuscular injections, port‐a‐catheter access, nasogastric tubes, mobilization) were excluded. The final sample comprised a total of 13 children aged 4–14 years. A total of 61 child‐clown interactions were observed. Each child was assigned an identification number (Table 4).

### 2.3 Data collection

The source of data collection was participant observation and informal interviews with children.^30^, ^31^, ^32^ Data were collected consecutively between October 30 and December 13, 2017. The observations lasted approximately four to six hours a day. Predicting how many children would be having a painful procedure or condition in the observation period was not possible; therefore, the decision was to sample all children interacting with the clown who met the study's inclusion criteria. Based on nurses’ knowledge of the individual child (e.g., plans for treatment and care, scheduled procedures), the nurses in the ward collaborated with the researcher by providing information on children that would potentially be included in this study. Sampling was convenience based on the specific experience of interactions between the child and clown judged to be of interest.^28^ Data collection was consecutive^33^ and conducted through focused ethnographic observations and informal interviews with all children during the recurrent encounters.

Over the entire data collection period, grand‐tour observations alternated with mini‐tour observations,^27^ because it was not known beforehand which children would be admitted on the days when the clown was present. However, the researcher followed the clowns on their scheduled days, observing, and interviewing children that met the inclusion criteria. The grand tour aimed to get an overview of the setting, the interaction with clowns, and how the ward was organized regarding the process with admission of children. The mini tour included the group of children followed over the entire fieldwork period with more than one encounter. The fieldwork focused on specific features such as place, actor, activities, object, time, goal, and feelings^27^ in the interaction between the child and clown during recurrent encounters. The researcher had an insider perspective based on prior work experience as a nurse in a similar cancer ward, which facilitated the contact with children, parents, and the healthcare staff. Observing the child‐clown interactions was considered the outsider perspective. Thus, the fieldwork involved a position between participation and observation that struck a balance between insider and outsider perspectives.^26^, ^27^ Conscious awareness of balancing between insider and outsider perspectives was established, for example, by wearing a nurse's uniform, assisting, and answering questions from children, parents, and nurses in an insider role. In other cases, the role of the outsider, as an observer at a distance, was predominant.

Informal interviews were conducted in a collaboration between child, clown, parents, and researcher immediately after the specific encounter or during the next encounter, by posing questions to the child referring back to the encounter, such as the following: “Tell me how you managed last time.” During informal interviews with children, the use of creativity in a childlike, age‐differentiated approach was regarded as enhancing the articulation of the child's perspective,^32^, ^34^ though the clown was in some cases involved in the dialogue concerning the articulation of the child's experience.

Handwritten scratch notes were completed during the fieldwork. Expanded field notes were prepared after the day's observation, using descriptions of speech and nonverbal behavior in order to facilitate the construction of the analysis process.^26^ These field notes were typewritten verbatim.^27^ In total, 75 hours of participant observations were conducted over 15 days, resulting in 43 pages of field notes.

### 2.4 Ethical considerations

The study was approved by the pediatric administration at the university hospital and by the Danish Data Protection Agency (Journal no. 2008‐58‐0028; id: 2016‐5). Approval from the ethics committee was not required according to Danish law. Consent was obtained from the clowns before data collection based on written and oral information about the study. Children and parents received information about the study, and their consent was obtained in the same session. All children, their parents, and clowns willingly participated.

### 2.5 Data analysis

The analysis explored child‐clown interactions during recurrent pain‐related procedures and conditions. The analysis was based on a qualitative thematic approach and unfolded as a dynamic process including five phases.^35^ Phase one comprised a thorough reading of the field notes to gain familiarity with the content. In phase two, initial coding was generated by organizing the entire data set into groups. In phase three, the research team was continuously involved in the sorting of codes and interpretation of the data, resulting in an overarching theme, two preliminary themes, and eight preliminary subthemes (Table 1). In phases four and five, a rereading of the entire data set was conducted. A review of the selected quotes was then conducted, discussed, and agreed upon in collaboration with all of the authors to ensure that the themes and subthemes fit in relation to data.^35^ Accordingly, the preliminary subthemes were refined, consequently decreasing from eight to four. Finally, returning to the data, one of the two preliminary themes was further reclassified from *For the next time* to *Hope in the WE*. Thus, a change in hierarchy level and labeling of themes and subthemes took place, which was legitimized by a thorough inspection of the chosen data extracts, illustrated in Table 1. Tables 2 and 3 illustrate the relationship among themes, subthemes, and codes of the two themes: *stronger in a WE* and *hope in the WE*.

**Table 1:** Illustration of analysis process

Preliminary overarching theme: *Hospital clown creating continuity in an ongoing WE*		Final overarching theme: *An ongoing WE*	
Preliminary themes	Preliminary subthemes	Themes	Subthemes
Stronger in a WE	Step by step/small steps	Stronger in a WE	“Maybe today…?”
	Providing a seat for the hospital clown		
	WE are all together		“You are my friend”
	The importance of having a friend		
	WE give high five		
For the next time	“You did it”	Hope in a WE	“WE did it your way”
	“See you again”		
	The way to do it		“See you again”

**Table 2:** Stronger in a WE. Relationship between theme, subthemes, and codes

Theme	Subthemes	Codes
*Stronger in a WE*	*Maybe today…?*	Clown asking for permission and using direct/indirect techniques
		Mutual small steps taken in establishing the relationship
		Child had the experience that the clown had enough time
		Child and clown asking for each other to meet
		Contact on the child's terms
	*You are my friend*	Clown providing the child a feeling of someone to share with
		Child explicitly sharing both the funny and the negative experiences and thoughts
		Mutual physical and mental contact
		Make and agree on plans together
		Mutual caring

**Table 3:** Hope in the WE. Relationship between theme, subthemes, and codes

Theme	Subthemes	Codes
*Hope in the WE*	*WE did it—your way*	Clown verbalizing how the child managed
		Child and clown having time to stay together in the evaluation
		Clown insisting on an evaluation
		Clown encouraging the child to discuss previous experiences
	*See you again*	Clown offering promise of another encounter
		A mutual expectation of meeting again
		Making deals for next time
		Planning the next scheduled procedure
		Giving high five

### 2.6 Validity and rigor

The researcher's insider perspective might have resulted in unconscious anticipation of the likely experiences. However, familiarity with the specific situations may also have facilitated the motivation to remain present in these emotional situations.^26^ To validate the interpretations of the participant observations, a form of member checking^35^ was performed in a dialogue with some of the children, parents, involved nurses, and clowns with a focus on their perspective on the interaction. This dialogue was conducted on the specific day of the child‐clown interaction or after the following encounter, respectively, to ensure a good fit between the researcher's and the participants’ view of the observed situation. Credibility was further enhanced by continuous involvement of the research team in the analysis and interpretation.

## 3 RESULTS

The overarching theme, *an ongoing WE*, was based on two themes and four subthemes (see Table 1).

Overall, the participant observations suggested that the child and clown were related in *an ongoing WE*, characterized by a strong and close relationship that developed between the child and clown over time during repeated encounters. The WE represent a responsive child/clown interaction evolved during a continuous, mutually focused attention.

Table 4 presents the demographics of the children and the number and characteristics of the encounters.

**Table 4:** Demographics and the number and characteristics of encounters

Child ID	Child characteristics (gender, age, diagnosis)	Encounter with a procedure or painful condition	Encounter without a procedure or painful condition	Total number of encounters with clown	Prior/new contact with clown	Clown ID
1	Boy, 5 years old, kidney tumor	3 (medication, intravenous cannulation)		3	New	A, B
2	Boy, 5 years old, brain tumor	1 (venipuncture)	1	2	Prior	A, B, C
3	Girl, 4 years old, leukemia	3 (mobilization, painful eating)	4	7	New	A, B, C, E
4	Boy, 13 years old, leukemia	3 (mobilization)	2	5	Prior	A, B, D, E
5	Boy, 8 years old, neuroblastoma	2 (intravenous cannulation)		2	Prior	A
6	Boy, 12 years old, leukemia	3 (nasogastric tube, mobilization, eating)	3	6	Prior	A, B, D, E
7	Girl, 13 years old, leukemia	3 (port‐a‐catheter access, intravenous cannulation)		3	Prior	A, C, D
8	Boy, 8 years old, leukemia	3 (mobilization, venipuncture, eating)	3	6	New	A, B, C, D, E
9	Boy, 6 years old, non‐Hodgkin lymphoma	5 (inhalation on mask, nasogastric tube, mobilization)	7	12	Prior	A, B, C, D, E
10	Boy, 14 years old, brain tumor	2 (examination, eating)	2	4	Prior	A, B, C, D, E
11	Boy 8 years old, Ewing's sarcoma	2 (subcutaneous injection, mobilization)	3	5	New	A, B, C, D
12	Boy, 12 years old, leukemia	2(venipuncture)	1	3	Prior	A, B
13	Boy, 4 years old, arthritis	1 (venipuncture)	2	3	Prior	A, C, D
Total	13	33	28	61	4 New9 Prior	5

### 3.1 Stronger in a WE

This theme concerns mutual interest and recognition, reflected by a responsive interaction between the child and clown. It covers the small, conscious steps taken by the clown when meeting the child in an attempt to establish a trusting relationship, tailored to the child's needs. *Stronger in a WE* is further specified in the two subthemes *Maybe today… ?* and *You are my friend*.

*Maybe today… ?* was demonstrated by the clown's cautious approach, beginning with the first encounter. The clown demonstrated an expectation that there would always be a new chance of meeting by asking “*Maybe today..?”* Thus, the clown invited the child into a WE on the child's terms and included the child in a negotiation of the encounter's content.

Initially, the clowns carefully asked for permission to enter the child's room, using direct or indirect contact (e.g., standing in the doorway without saying anything, asking directly whether the child wanted a visit) and waiting for the child's consent. This cautious approach was found whether it was the first encounter between child and clown or whether they had met before. The clown tailored the strategies depending on the individual child's situation and condition. Acknowledging the current state of the child sometimes resulted in postponing the encounter (e.g., the child asked the clown to return later that day or another day; the clown just said *hello* and went back later). Otherwise, the clown's cautious approach helped the child to place the pain in the background and to consequently be distracted from or not pay attention to the pain. This was illustrated in a field note regarding a 13‐year‐old boy suffering from pain in his legs (and lying in bed): *The clowns asked in the doorway, “Do you want us to come in?” The boy nodded. The clowns started a fantasy play* [giving different roles to each other in a kind of game] *with the mother. After a few minutes, the boy became a part of it. The play went on for 20 minutes. Then the clown (A) said thoughtfully, “Okay, how is it to be the real* [child's name] *today?” The boy said, “Hard!” There was silence in the room, and the clown looked intensely at the boy, saying, “I know you like the table football, I see your pain, but… anyway… I'd like to ask if you would like to play with me?” There was a pause, and then the clown said, “If not today—maybe tomorrow?” The boy looked up with a big smile and said, “Yes, mum, find my shoes, I am ready!”* (ID 4).

Specific knowledge of the child's preferences helped the clown establish trust during the initial encounters, which in turn helped the child share their thoughts and feelings. Often, clowns gave the child an experience of another's presence simply by spending time with them. The child often asked for an encounter related not to a specific procedure but rather to a generally painful or sad situation. In response, the clown showed through an empathetic attitude that the situation of the child was understood. Such an interaction is illustrated by the field notes regarding a 12‐year‐old‐boy: *His mother said, “He has had a really bad day with the plan for a new nasogastric tube—so much pain. He is very sad—he does not want to either eat or talk, but he has asked for you* [the clown]. *The clown (A) knocked on the door and showed his head in the doorway. Without words, the boy nodded toward a chair* [for the clown]*. The clown, who appeared sad and looked in the boy's eyes… let the time go… The boy whispered, “This is a fucked‐up disease*.” *There was a long pause, and then the clown said, “Should I be here tomorrow as well?” The boy asked, “Can you help me with the tube?”* (ID 6).

In the examples presented above, parents seemed to play an integral role; they were either explicitly involved by the clowns or became involved on their own initiative. In some cases, the clown asked the parents in advance about the condition of the child or the parents informed the clown about the child's condition in the hallway, which helped the clown take a cautious approach, asking *Maybe today… ?*

The subtheme *You are my friend* refers to involvement, closeness, and interest, expressed both verbally and physically by the child and clown during the recurrent interactions. Both children and clowns used the word *friend*. Most children asked expectantly for the clowns to come visit them. Children and clowns showed that they cared about each other, demonstrated physically in hugs, high fives, holding hands, etc. and emotionally in eye contact and intense gazes. With the clown present in an interaction, the child achieved a feeling of sharing the pain and challenges, hence easier management of the pain‐related procedure or condition. The relationship resulted in a mutual expectation and hope for support from the clown in an ongoing relationship.

The relationship assisted the children in managing the situation. The child's ability to collaborate in pain‐related procedures and conditions was thereby strengthened. Specifically, children's responses to the clowns took the form of relieved and joyful body language, as exemplified in the observation of a 12‐year‐old boy with prior negative venipuncture experiences: *One of the clowns put his arm around the boy's neck and said, “Now I see you are worried, my friend”? The boy nodded, looked with an intense gaze on the clown, and cried. The clown said, “I can follow you, and WE can help each other to manage the venipuncture, if you like”? The boy looked up with a little smile, saying, “YES.” The clown smiled and said, “And then WE will bring in your mother, she is good at holding your hands—right, Mum? And I will stay with you the whole time.” The boy maintained an intense gaze on the clown and grasped his hand* (ID 12).

Additionally, parents verbally expressed that the clowns helped their child like a friend. A mother said, *“They* [the child and clown] *are so important to each other… especially in the procedures… they really see each other as best friends”* [ID 5].

Children alternated between different emotional states, depending on the painful condition or situation. In some situations, children expressed relief when the clown was present, which overshadowed the unpleasant feeling; in other situations, the child was sad and worried. In response, the clown tailored the approach to the child and interacted in play experiences and dialogue based on the child's expressions and initiative. This was observed during two encounters with a 4‐year‐old girl. She had been a little hesitant and, when she first met with the clowns, observed them at a distance: *She saw the clowns in the hall area and ran straight into their arms. The clowns said, “Hello my friend, nice to see you.” They gave the girl a big hug, and she immediately started telling stories with a smile all over her face. “I want to share a cookie with my friends, come to my room!” A few days later, she was in bed, having great problems eating due to pain in her mouth. The clowns began doing funny exercises on the floor without directly involving the girl. The clowns kept an eye on her. Tears ran from her eyes, and she looked with hesitant interest from her bed. During the play, the clowns ate pieces of chewing gum and showed with exciting sounds how they enjoyed the taste. Suddenly the girl started to eat pieces of orange one by one, while she looked with a smile toward the clowns. Her mother said, “Fantastic! She had not eaten for several days; you* [clowns] *are her friends”* (ID 3).

In summary, *stronger in a WE* indicates the responsive interaction in which the clown gradually built up a relationship with the child from the first encounter (*Maybe today… ?*), achieved knowledge of each individual child, and created continuity over recurrent encounters. When a contact was established between the child and clown, the relationship was eventually taken for granted by children, parents, and the clown, expressed as the child's expectation to be with the clown (*You are my friend*) and assuring the clown to help the child to manage.

As a result of the clowns’ approach of taking small steps based on the child's behavior and vocalization, a responsive interaction (WE) evolved over time between the clown and the child. During the WE, the clown encouraged children to become active participants, and express thoughts and feelings. This facilitated a feeling of closeness and led to a strong, supportive relationship, which was understood as friends caring for each other and sharing their feelings. This relationship enhanced the child's ability to manage the painful procedures and conditions based on a feeling of being *Stronger in a WE*.

### 3.2 Hope in the WE

The overarching *ongoing WE* theme focused especially on hope, which was related both to current and future painful experiences. Moreover, the child's hope was related to the belief that the clown would help them in future encounters and the desire for the clown to do so. The recurrent feature of the relationship was that the child and the clown cared about each other, which gave the child a feeling of not being alone during the painful procedures or conditions. The hope was promoted by an evaluation of the situation and of the child's reaction, which created awareness of the child's individual strategies. Hope in the WE was divided into two subthemes *WE did it—your way* and *See you again*.

The *WE did it—your way* subtheme reflects the trusting moments, in which the focus was on the child's individual strategies for managing a specific situation. The clown's use of intuitive strategies based on a deep insight into the individual child's history and a situational understanding of the child's needs fulfilled the child's wish for help. The clown initiated an evaluation that generated a mutual understanding and clarification of the child's resources for managing the painful situation. This was observed in an 8‐year‐old boy, who had previously met the clowns twice with a hesitant expression: *The boy was in bed with pain from a central venous cath. He had a worried expression on his face. The clowns arrived at the moment when the nurse was to begin the procedure. The boy said, “I hate the smell of the alcohol.” One of the clowns said, “Please stop for a moment.” The clown ran out and came back after a few minutes with some vanilla in a bowl. The boy grabbed the bowl with vanilla and managed the procedure by smelling it. Then he spontaneously grabbed a chocolate bar from the pocket of the clown and started eating. The clown said, “You DID it, you DID it by smelling the vanilla and eating our chocolate bar—remember this for the next time.” The clown focused his eyes on the boy. The boy looked at the clown and nodded eagerly. Then the clown looked at the boy's parents, saying, “Remember this, mother and father, and buy these chocolate bars for the next time”* (ID 8).

*WE did it‐your way”* provided the child with a strategy for managing painful situations in the future, and the clown continued with the tailored approach to make the child aware of the individual strategies the child had successfully used. This was illustrated by an explicit mutual wish for another meeting, observed in a 12‐year‐old boy, who was scheduled to have a nasogastric tube inserted. He had asked the clowns to accompany him during the procedure: *One of the clowns said, “You have managed before. WE know your way. WE can manage together.” The other clown looked and nodded, with a serious gaze at the boy. The boy said, with tears running, “I scream! I want music and your hand.”* [The tube was inserted.] *Afterward the clown and the boy sat together, still holding hands. The clown said, “You managed, you did! This was the third time.” The boy nodded, saying, “I want you to be with me every time,” and tears continued to run from his eyes. The clown said, “Yes WE can do it together like this time and make your own plan”* (ID 6). This observation was an example of how the children felt assured during the interactions that the clown could be present and support them in future as a friend. Moreover, the children did not talk much about the pain itself; they rather shared their feelings about the ways how to manage it.

The subtheme *See you again* was expressed by children in multiple ways as a wish for help during their next procedure and as a more general hope to spend time with the clowns. This was observed in a 13‐year‐old boy lying in bed with fever and pain, who had played table football with the clowns once before: *The clown walked into the room, positioned himself at the edge of the bed, and looked at the boy, saying nothing. After a minute, the boy said, “I hate this situation… cannot stand on my legs today… “The clown looked intensely at the boy. The clown said, “I hope we will play another football game.” Then there was a long pause, and the boy answered, “Me too.” The boy raised his hand and gave the clown a high five, saying, “WE can do next time—hopefully I will be better next time… “*(ID4).

Children felt reassured in their belief that the clown would come again, and a specific strategy with the clown was often included in the child's wish for the next encounter. Additionally, clowns always expressed an explicit wish to meet the child again by ending the encounter with *“See you again,”* providing the child with hope. This was exemplified by an encounter with an 8‐year‐old boy, who was undergoing a subcutaneous injection: *When the subcutaneous injection was done, the clown said, “What about the port‐a‐cath access tomorrow? I heard from your mother that this is the worst thing for you.” His mother nodded, and the boy said, “See you again tomorrow, and WE can play the song I like—the song we did today. Let's do this tomorrow”* (ID 11).

As illustrated, the clowns facilitated the engagement of parents as partners. This was done by explicitly including parents in the encounters by verbalizing their role and, in terms of planning for the future, by asking them to become a part of the mutual plan to help their child (e.g, buy the chocolate bar; ID 8).

*Hope in the WE* refers to a mutual expectation and belief that the child and clown will meet again (*See you again*). This expectation and belief was conveyed by a relieved expression of hope, from the child and clown, for another encounter. The hope was supported during an evaluation of the current situation, pointing to the child's ability to manage future painful situations (*WE did it your way*).

Overall, the creation of *hope in the WE* showed the important role of the clown in maintaining a focus on the needs and the expressions of the individual child. The hope was invariably related to the child's and parent's expectations of help from the clowns, when managing current pain‐related situations and future meetings. The children felt reassured during the interactions that the clown could be present and support them in the future as a friend. During an evaluation by the clown in which the clown actively involved the child, the hope for managing the next pain‐related situation was made possible and continuity in the management strategies was ensured.

## 4 DISCUSSION

The aim of this study was to explore the characteristics of the interaction between the child and hospital clown during recurrent hospitalizations for repeated pain‐related procedures and conditions. The study showed how interactions between the child and clown in *an ongoing WE* fostered a continuous, trusting relationship. The relationship was reflected in two themes: *Stronger in a WE* and *Hope in the WE*. This WE was built up based on specific knowledge of the individual child's preferences, situation and earlier shared experiences. The findings suggest that the *ongoing* WE assisted the child in managing the situation and resulted in a mutual expectation which provided the child with hope for a continuous relationship. Moreover, strengthened the child's ability to manage painful events and facilitated the experience of hope for managing future painful events. Specifically, children and parents experienced the clown as a friend who cared for and supported the child by instilling positive expectations regarding the child's approach to managing current and future painful events.

The importance of establishing a trusting relationship by taking small steps from the first encounter tailored to the individual needs of each child was illustrated in the subtheme *Maybe today… ?* By this question, the clown demonstrated an expectation that there would always be a new chance of meeting. These findings are in line with those of Tener, Ofir, Lev‐Wiesel, Franco, and On^36^ who found a positive effect on 5‐ to 16‐year‐old children's experience of an invasive examination when they were accompanied by a clown and underlined the importance of the clown in building trust even in one encounter. The current findings also support the work of Linge,^21^ who interviewed nine children (3–18 years) hospitalized for various lengths of time and found increased self‐confidence and a feeling of well‐being during recurrent interactions with hospital clowns. However, most research on clowns’ impact during painful procedures is focused on short‐term procedures.^4^ Thus, time may be a factor that influences the clown's ability to establish a therapeutic relationship within a context of short encounters with a limited time frame for preparation.^37^ Nevertheless, knowledge of the individual child is essential to establishing a trusting relationship. Accordingly, the first moment of the encounter can lay the foundation for a supportive relationship.^36^ This study offers insights into the importance of establishing and maintaining a continuous, trusting relationship between the child and clown.

The *You are my friend* subtheme revealed that the child‐clown relationship develops on the basis of mutual acknowledgment and a wish to share almost everything as friends. The contact established over time had the character of a friendship, expressed physically and verbally by the child, clown and parents. However, despite the use of the term *friend*, the relationship between the child and clown is still professional from the clown's perspective. The clown set the direction by preparing the child to manage painful situations independently in the future, which is an expectation for the professional relationship.^12^ What is unique in the professional context with the clown is the perspective that the encounter is entirely on the child's terms.^18^, ^25^ Thus, the current findings indicate that this professional relationship contains elements of friendship, such as mutual care and the development of interpersonal closeness.^38^ Elements of friendship were also reported by Kristensen et al.,^19^ who found that a WE characterized by a familiar atmosphere strengthened 4‐ to 15‐year‐old children's competence in pain management and coping experience during a one‐time acute venipuncture procedure. In a study by Ofir et al.,^25^ children aged 5–16 years described the relationship with clowns as friendships based on elements of sharing, listening, and companionship. The findings of our study are consistent with those of Ofir et al.,^25^ who found that the specific term *friend* was used by both children and clowns as a technique of distraction and means of expressing support and care in a trusting relationship. Our findings contribute nuance to the existing evidence by demonstrating the positive impact of a continuous, supportive, and professional relationship with elements of friendship on the child's ability to cope with future painful events.

The subtheme *We did it—your way* reflects an evaluation performed by the clown, which seemed to strengthen the child's confidence in his or her ability to manage painful situations. The evaluation focused on the individual strategies used by the child and the plan for future encounters, which created the basis for the child's *hope in the WE* as a future resource for coping. Our findings suggest that it is important for children to receive focused attention from clowns or nurses in order to maintain the hope that a given procedure is going well. Current research^19^ had underlined the importance of an evaluation with the clown to create an awareness of the child's individual coping strategies after acute venipuncture. Consistent with our findings, Tener et al.^36^ showed that parents report a continuing positive effect of clowns after the return home, describing the clown as part of a coping strategy that remained useful in the future life of the child. The importance of evaluating how the child coped with a painful procedure is supported by research on memory reframing,^10^, ^39^ which has shown that more accurate memories of pain are linked to reduced pain and distress during subsequent pain‐related procedures. Thus, the tailored evaluation phase might offer an opportunity for positively reframing the child's memories.

Every child experiences painful procedures and conditions in a different manner.^40^ Additionally, the coping process is constantly changing and is thereby influenced by the child's previous experiences.^12^ Thus, there is a need to be aware of the child's prior experiences, stressed in this study as the importance of a continuous relationship in a trusting, ongoing WE. The process of evaluation created awareness of the individual coping strategies, which seemed important for the child and were implemented in the following pain‐related situations. In particular, *hope in the WE* presented during this evaluation may strengthen the child's competence in managing painful procedures and conditions.

Although children expressed worries concerning the future, they were helped by the identification of strategies compatible with the planned procedure and the certainty of the clown's presence in the future (expressed as “see you again”). The hope was expressed both as a specific wish for the clown to be present during the next procedure and as a more general hope for sharing important moments related to the pain‐related challenges. The ongoing child‐clown relationship and the influence on hope for the future has not been thoroughly investigated to date. However, Linge^21^ demonstrated that the relationship with clowns over various lengths of time offered hope for making things easier. Children in the current study were confronted with painful events, which might seem hopeless. Hope involves an attainable desire and is related to trusting dependence on others.^38^ Accordingly, children in this study expressed hope that the support of the clown would continue. Hence, our findings reinforce the importance of the *ongoing WE*, in which hope is closely connected to the child's experience of knowing how to manage painful situations now and in the future. In addition, the results concerning the importance of establishing *an ongoing WE* add to previous findings on establishing a WE during acute venipuncture.^19^

### 4.1 Recommendations for improving nursing practice

The expressions of the child need to be heard and integrated into care for the child from the first encounter and maintained throughout subsequent encounters. The present findings underline the importance of establishing a continuous, trusting relationship in *an ongoing WE* on the child's terms, which can serve as a resource for improving the management of pain during painful procedures and conditions. Nurses can advocate for the involvement of clowns during procedure‐related pain and conditions for recurrent hospitalized children. In addition, the approach and techniques used by the clown in establishing and maintaining *an ongoing WE*, represented by a continuous, responsive interaction, can inspire nurses to improve the child's management of pain‐related procedures and conditions. Given the opportunity and time to establish a relationship with children undergoing these procedures and conditions on a recurrent basis, clowns can work together with nurses in caring for such children.

### 4.2 Limitations

Only one ward was chosen for data collection, and this limited the number of children included in the study. Hence, a replication of this study with more children in different or similar conditions could have contributed nuance to the findings. Moreover, a longer period of fieldwork combined with other methods, such as interviews with parents or nurses could have enriched and added nuance to the findings on how the child‐clown relationship evolved over time and influenced the experience of pain. The inclusion of a pain intensity measure may have enhanced the results (i.e., demonstrating change in pain scores over the encounter), however, was not done due to the type of interaction between child and clown. These perspectives were not included in this study and might have limited its insights, particularly regarding the underlying role of parents. Finally, interviews with the children and hospital clowns after or at the end of the study period could have provided insights into the long‐term influence of the interaction with the clowns represented during an ongoing WE.

Participant observation can offer knowledge about children that cannot be obtained adequately through other methods.^41^ However, video recording might have captured more details of the child‐clown interaction.^42^ Nevertheless, children with recurrent hospitalizations are a vulnerable group. An overriding concern for addressing the research question with minimal risk for the child and family^41^ was the reason for not including video recordings.

## 5 CONCLUSION

This study has identified the meaning of *an ongoing WE* established with children during recurrent pain‐related encounters with hospital clowns. The clown was found to be an important and close professional friend, who developed a trusting relationship using specific knowledge of the child's preferences, situation, and earlier shared experiences. This relationship mitigated the child's feeling of being alone and thereby positively contributed to the child's ability to manage painful situations. The clowns helped children to identify, practice, and implement individual strategies for coping with specific painful situations. Reflection on and evaluation of how the child managed the situation may have influenced the child's approach to managing future procedures. Importantly, the evaluation focuses on building competencies and providing the child with a feeling of hope for managing future situations.

This study provides useful insights into establishing a continuous, trusting relationship, represented in the overarching theme of *an ongoing WE*. The establishing and maintenance of an ongoing WE may improve the psychosocial care and management of pain for children undergoing recurrent pain‐related procedures and conditions during recurrent hospitalizations. Thus, these findings may support and advance nursing care associated with procedure‐related pain management. In addition, the study suggests avenues for multidisciplinary initiatives, such as nurses’ advocacy for the inclusion of clowns during recurrent interactions as a means of ensuring best practices in managing recurrent pain‐related procedures and conditions. Further research is required to investigate the long‐term impact of clowns in this context, as well as the roles of parents and nurses in supporting child‐clown interactions in painful situations.

## CONFLICT OF INTEREST

The authors have no conflicts of interest to declare.

## FUNDING

This work was supported by the Danish Child Cancer Foundation, Copenhagen; the North Denmark Region Health Science Foundation; and the Clinical Nursing Research Unit, Aalborg University Hospital, Denmark.
